# Frustrated Lewis Pairs in Heterogeneous Catalysis: Theoretical Insights

**DOI:** 10.3390/molecules27123734

**Published:** 2022-06-10

**Authors:** Qiang Wan, Sen Lin, Hua Guo

**Affiliations:** 1 State Key Laboratory of Photocatalysis on Energy and Environment, College of Chemistry, Fuzhou University, Fuzhou 350002, China; n181310044@fzu.edu.cn; 2 Department of Chemistry and Chemical Biology, University of New Mexico, Albuquerque, NM 87131, USA

**Keywords:** heterogeneous catalysis, frustrated Lewis pairs, hydrogen dissociation, alkynes hydrogenation, density functional theory

## Abstract

Frustrated Lewis pair (FLP) catalysts have attracted much recent interest because of their exceptional ability to activate small molecules in homogeneous catalysis. In the past ten years, this unique catalysis concept has been extended to heterogeneous catalysis, with much success. Herein, we review the recent theoretical advances in understanding FLP-based heterogeneous catalysis in several applications, including metal oxides, functionalized surfaces, and two-dimensional materials. A better understanding of the details of the catalytic mechanism can help in the experimental design of novel heterogeneous FLP catalysts.

## 1. Introduction

**Figure 1:**
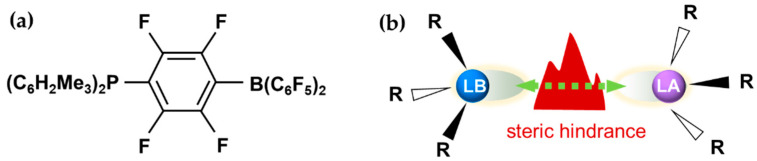
(**a**) Structure of the first reported homogeneous FLP. (**b**) Scheme of the common strategy for constructing homogeneous FLP catalysts. (**a**) is reproduced from reference [24] with permission.

Noble-metal-based catalysts have been an indispensable part of modern industrial catalysis [1,2,3,4]. However, their high cost and geopolitical risks have always placed a severe constraint on their applications. Considerable efforts have been made to reduce [5,6,7] and/or eliminate the use of noble metals in catalysts [8,9,10]. The former approach has greatly benefited from various strategies and concepts recently developed to improve the atomic efficiency and reactivity of active sites, such as alloyed catalysts [11,12,13], atomic dispersed catalysts [14,15,16], and surface defect engineering [17,18,19]. Meanwhile, significant efforts have been made to achieve similar activity to noble metals by utilizing other materials (e.g., earth-abundant transition metals and main-group elements) [20,21,22,23]. One such strategy is using frustrated Lewis pairs (FLPs), which have attracted much recent attention thanks to their superior performance in homogenous small molecule activation [20,24,25]. As shown in Figure 1, an FLP assembles a Lewis acid (LA) and base (LB) pair in proximity, but with steric hindrance to prevent them from recombining, so as to activate small molecules [26,27,28]. Besides catalyzing heterolytic H_2_ dissociation [29,30], FLP catalysts have also been used in the activation of other molecules (e.g., alkenes, aldehydes and CO_2_) [31,32,33,34,35,36,37], thereby providing a new strategy for synthetic chemistry. Inspired by unique FLP-based catalysts in homogenous catalysis, this concept has recently been extended to heterogeneous catalysis [38,39].

Solid surfaces provide a template for designing FLPs, which can be used to aid heterogeneous catalysis [40,41,42]. An early example involved the γ-alumina-catalyzed low-temperature activation of CH_4_ by Wischert et al. [43], who identified a surface FLP made up of LA and LB sites. The former was the tri-coordinated Al_III_, and the latter was O. The dissociation of CH_4_ led to Al–CH_3_ and O–H. In another case, Shi et al. observed chain structures on the ZnO(10$\bar{1}$0) surface [44], formed at an extremely low temperature (~20 K) by the heterolytically dissociative adsorption of H_2_, leading to a zinc hydride and a hydroxyl. Density functional theory (DFT) calculations found two kinds of Zn–O pairs on the surface. One has a bond formed between the Zn and O sites, which can be classified as classical Lewis pairs (CLPs). The other type of Zn–O pair has the Zn and O sites well separated, thereby forming FLPs. DFT calculations suggested that the H_2_ dissociation on the FLP is more favorable than on the CLP. Similar FLPs have been found on ceria (CeO_2_) [45,46], which was reported recently to catalyze the selective hydrogenation of unsaturated hydrocarbons [47]. In this case, the FLP, formed between a Ce^3+^ (LA) created near an oxygen vacancy (O_v_) and a nearby oxygen (LB), is shown to catalyze the heterolytic H_2_ splitting [46]. These surface species have indeed been identified experimentally [48,49], confirming the hypothesis. DFT calculations demonstrated that these species catalyze the subsequent hydrogenation [46].

Driven by these early successes, a large number of theoretical studies have recently been carried out to gain insights into heterogeneous FLP catalysis and to predict new heterogeneous FLP catalysts. The LA and LB sites that make up heterogeneous FLPs may be the electron-deficient and electron-rich atoms and groups externally introduced, or sites with different electronic properties constructed by surface engineering. Among the proposed heterogeneous FLPs, metal-organic frameworks (MOFs) and modified metal surfaces have been studied via density functional theory (DFT) calculations [50,51,52,53], and those based on two-dimensional (2D) materials and metal oxides have started to attract theoretical attention [17,54,55,56]. It is easy to understand why MOFs are good candidates for FLP-based catalysts, as various functional groups can be designed within a MOF with desired distances. On the other hand, metal oxides are naturally rich in LA (metals) and LB sites (oxygens), and surface vacancies can be leveraged in designing FLPs. The designs of FLPs on 2D materials are even more versatile. A typical practice is to use electron-deficient and electron-rich atoms to change the local environments [54,55,57]. These studies have greatly advanced our abilities to activate small molecules with surface FLPs. However, experimental conditions are often more complicated than theoretical models. Although several strategies, mechanisms, and structure–reactivity relationships have been proposed based on these theoretical studies, there is still a lack of consensus on how to design highly efficient FLP catalysts.

Here, we review the recent advances in theoretical studies of FLPs at gas–solid interfaces and discuss their roles in heterogeneous catalysis. We strive to survey the recent theoretical studies of heterogeneous FLP catalysis, aiming to classify various types of heterogeneous FLP catalysts and strategies for creating surface FLPs. We also discuss mechanisms and structure–reactivity relationships that could potentially assist researchers in the rational design of the corresponding catalysts.

This review is organized into four sections. In Section 2, we briefly introduce the original concept of FLPs and the mechanism for small molecule activation by homogeneous FLP catalysts. Several FLP-based heterogeneous catalysts are discussed in Section 3. In Section 3.1, we present our current understanding of several metal-oxide-based FLP catalysts with the relevant catalytic mechanisms. Section 3.2 focuses on the progress of FLP-facilitated catalysis on functionalized surfaces, including MOFs, metal surfaces, and 2D materials. Finally, design strategy, challenges, and outlook are discussed in Section 4.

## 2. Mechanisms of FLP Catalysis

H_2_ activation has traditionally been catalyzed by noble metals. In 2006, Stephan and coworkers opened the door to metal-free activation of H_2_ by introducing an FLP-based homogeneous catalyst (C_6_H_2_Me_3_)_2_PH(C_6_F_4_)BH(C_6_F_5_)_2_ [24], which is shown in Figure 1a. A common form of the FLP consists of an electron-deficient atom (or group) and electron-rich atom (or group) in one molecule or in a molecular pair, which are prevented from neutralizing by utilizing geometry and/or steric hindrance. The role of the FLP is to promote H_2_ dissociation into protonic (H^+^) and hydridic (H^−^) species. Since then, homogeneous FLP catalysts have been developed to activate other molecules such as N_2_ and CO_2_ [34,58], and much effort has been devoted to the understanding of the mechanism [59,60,61].

**Figure 2:**
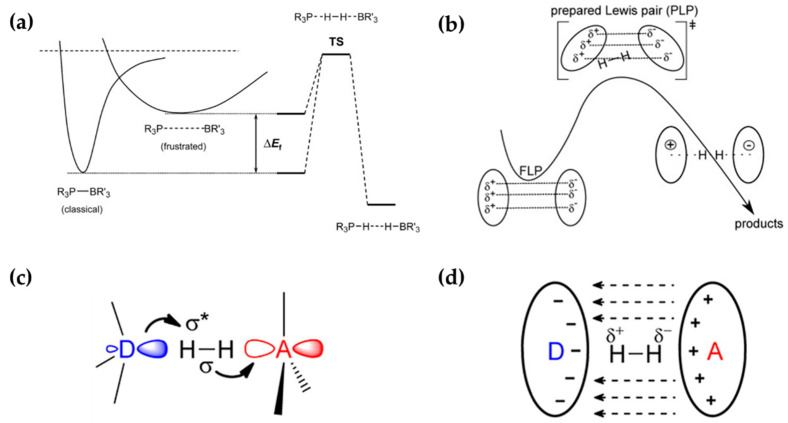
Role of (**a**) frustration and (**b**) electron field in mechanisms of H_2_ activation catalyzed by FLPs proposed by Rokob et al. and Grimme et al., respectively. Schemes of FLP-catalyzed H_2_ dissociation via (**c**) the ET mechanism (symbol * indicates antibonding orbital) and (**d**) the EF mechanism. (**a**), (**b**), and (**c**,**d**) are from references [62,63,64] respectively, with permission from John Wiley and Sons and American Chemical Society.

Theoretical studies have provided valuable insight into the mechanism of FLP-catalyzed H_2_ dissociation. The importance of the “frustration” between the LA/LB sites, namely, their spatial/steric separation, was confirmed by a theoretical study of Rokob et al. [62]. The resulting FLP differs from its classical counterpart, CLP, which is stable with low catalytic activity, by creating a pre-organized and strained environment for catalysis, thereby lowering the activation energy for hydrogen splitting, as shown in Figure 2a. Specifically, these authors attributed the catalysis to a covalent transition state involving electron transfer (ET) between the LA/LB pair and the H_2_ molecule. However, later theoretical studies by Grimme et al. [63] found little evidence for an activated complex LA–H–H–LB and the associated lowered barrier. Instead, the H_2_ molecule inserted into the FLP cavity dissociates spontaneously, and the activation is essentially the energy cost for H_2_ to enter the electric field (EF, Figure 2b) generated by the FLP; see Figure 2b. Both the ET and EF mechanisms, which are illustrated in Figure 2c,d, are probably operative to some extent, but their importance might depend on the specific system [64].

The situation in heterogeneous FLP catalysts is expected to be similar to that of its homogeneous counterpart. The possible mechanisms and corresponding applications of FLPs in heterogeneous catalysis are discussed in detail in the following sections.

## 3. Current Developments in Heterogeneous FLP Catalysts

### 3.1. FLPs Based on Metal Oxides

Metal oxides, possessing natural LA and LB sites on their surfaces, are known as heterogeneous catalysts for C–H and H–H bond activation. Studies suggested that the Lewis acid/base properties of the active sites on metal oxide surfaces could provide a unique way to obtain an in-depth understanding of the catalysis [65].

**Figure 3:**
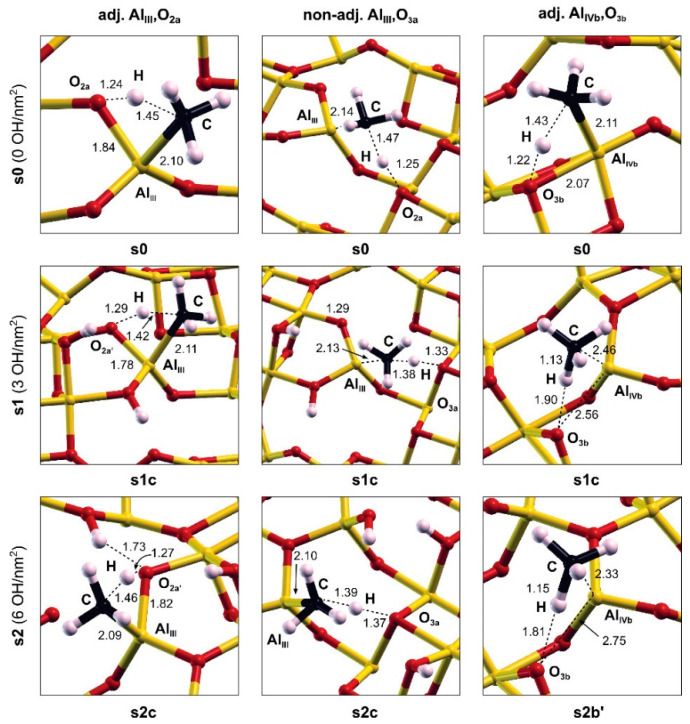
Configurations of the transition states for CH_4_ dissociation on various surface Al–O pairs for surfaces at different OH coverages (0, 3 and 6 OH/nm^2^). Adapted from reference [66] with permission.

Λ-alumina (γ-Al_2_O_3_) is considered as a heterogeneous FLP catalyst for C–H bond activation in CH_4_. In a combined experimental and first-principles study, Wischert et al. revealed that low-temperature heterolytic C–H bond cleavage in CH_4_ on a hydroxylated γ-Al_2_O_3_(110) to form Al-CH_3_ and O-H can be ascribed to the co-action of non-adjacent Lewis acid–base pairs consisting of surface Al_III_ (LA) and O (LB) sites [43]. These two sites are separated by 4.1 Å on the hydroxylated alumina surface, forming a cavity for the adsorption and activation of CH_4_, leading to Al–CH_3_ and O–H. They also speculated that such FLP catalysis might be general for different oxides towards other molecules with polarizable X^+^–Y^−^ bonds. In the subsequent study by the same group, the authors theoretically compared the C−H or H−H bond cleavage of CH_4_ or H_2_ catalyzed by a frustrated Al–O pair over the γ-Al_2_O_3_(110) and γ-Al_2_O_3_(100) surfaces as a function of hydroxyl coverage [66]; see Figure 3. Similarly to the case of CH_4_ activation, the activation of the H–H bond in H_2_ leads to Al−H and O–H species. The activation of H_2_ is more facile because of the lower dissociation barrier than that for CH_4_ activation. Importantly, these studies demonstrated that the reactivity of Al and O sites is affected by partial hydroxylation, which stabilizes the metastable (110) surfaces and tunes the electronic structures of the Al–O FLP by increasing the acidity of Al and the basicity of O. Hence, surface hydration can be used as an effective strategy to create and stabilize surface FLPs on (nonreducible) metal oxide surfaces.

Another example of FLP-facilitated heterolytic dissociation of H_2_ was reported to occur on hydroxylated indium oxide (In_2_O_3−x_(OH)_y_) which catalyzes the reverse water gas shift (RWGS) reaction (CO_2_ + H_2_ → CO + H_2_O). Starting with the H_2_ activation on the surface, two dissociated H atoms and one of O atoms from CO_2_ molecules combine to form a H_2_O molecule, leaving CO. In DFT calculations of this reaction combined with in situ spectroscopic and kinetic studies by Ghuman et al. [67], the activation of H_2_ was attributed to surface FLPs consisting of non-adjacent hydroxide (LB) and indium (LA) pairs, and the later are exposed at a surface oxygen vacancy. The proposed mechanism leads to the formation of surface O–H_2_ and In–H species, which subsequently attack the adsorbed CO_2_ to produce CO and H_2_O.

**Figure 4:**
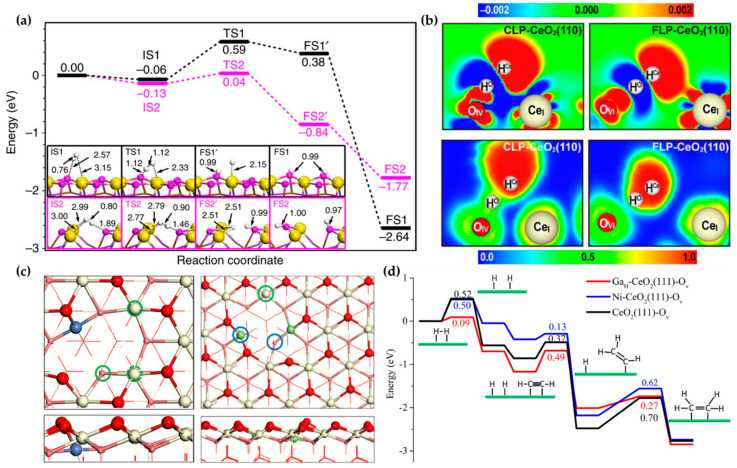
(**a**) Calculated energy profiles of the FLP (magenta line) and CLP (black line) catalyzed H_2_ dissociation of CeO_2_(110) with (magenta line) and without (black line) O vacancies, based on PBE via VASP. Color code: yellow, Ce; pink, O; white, H. (**b**) CDD (upper) and ELF (bottom) maps of H_2_ activation TS on CeO_2_(110). (**c**) Top and side views of Ni(left)/Ga(right)-doped CeO_2_(111). FLPs are marked by solid circles. Color code: yellow, Ce; red, O; blue, Ni; green, Ga. (**d**) Energy profiles of acetylene hydrogenation catalyzed by Ga/Ni-doped CeO_2_(111) and defect CeO_2_(111), based on PBE-D3 via VASP. (**a**–**d**) were adapted from references [45,46,73,74], all with permission.

The existence of FLPs is of course not restricted to the two examples discussed above. Ceria (CeO_2_) has long been used as a support for metal catalysts, thanks to its strong reducibility [68]. The recent discovery by Vilé et al. that ceria alone is capable of catalyzing selective hydrogenation of unsaturated hydrocarbons [47,69,70] has stimulated considerable interest in understanding its reactivity and selectivity [71]. The initial proposal of the mechanism for partial acetylene hydrogenation contained a relatively high barrier (2.86 eV) [69], which is kinetically unattainable. The key flaw of that mechanism was the assumption of homolytic activation of H_2_, leading to the formation of two surface OH species, which are responsible for the high barrier in the hydrogenation step. Interestingly, other DFT studies showed that heterolytic activation of H_2_ has a significantly lower barrier [72]. In this case, an exposed Ce serves as the LA for accepting the hydride (H^−)^ species from the dissociating H_2_, and a surface O can serve as the LB for the proton (H^+^). Subsequent DFT calculations revealed the crucial role of O vacancies in forming the surface FLPs [45,73], and mechanism investigation suggests that the energy barrier of the rater-determining step of acetylene hydrogenation catalyzed by the FLP is lower than that of a CLP on CeO_2_(110) facets (Figure 4a). One can see from the charge density difference (CDD) in Figure 4b that in the TS of H_2_ activation, electron density is increased around H on Ce and decreased around H on O with both CLP and FLP. Meanwhile, it can be inferred from the electron localization function (ELF) maps that there are some highly delocalized regions between H^-^ and Ce, and the region between O and H^+^ shows a strong covalent property. All these suggested that both the local electric field on the surface and the electron transfer between H_2_ and FLP/CLP contribute the reactivity. Based on our DFT calculations on CeO_2_(111), the proton and hydride species are crucial for the subsequent hydrogenation steps [46,74], leading to a mechanism that is consistent with experimental observations [49]. All the above studies suggested that the surface FLPs are crucial for the heterolytic H_2_ dissociation. Recently, Wu et al., using in situ inelastic neutron scattering spectroscopy (INS), revealed that the hydride (Ce–H) species is indeed present during the acetylene hydrogenation catalyzed by ceria [48]. In the meantime, Moon et al. detected surface OH species on the ceria catalyst using in situ diffuse reflectance infrared Fourier transform spectroscopy (DRIFTS) [75]. These experimental data thus provided strong supporting evidence for the heterolytic dissociation mechanism catalyzed by surface FLPs.

Doping has been shown to enhance the FLP’ catalytic activity on ceria. For example, Ni doping promotes oxygen vacancies (Figure 4c), leading to effective Ce/O FLPs [46,76] without directly participating in catalysis. Doping of Ga can also lead to novel single-atom catalysts in which the LA is provided by the doped metal on ceria surfaces [74], and results in a lower barrier for H_2_ activation and more active H species, as illustrated by Figure 4d. Other dopants include copper [77,78]. DFT results show that atomic Cu promotes the formation of O_v_ [71], leading to Cu/O FLPs catalyzing the cleavage of the H–H bond. These Ce–O FLPs were also found to be active for CH_4_ activation, thanks to the strong interaction between the CH_4_ molecule and FLPs. In addition, these FLPs were shown to efficiently catalyze the non-oxidative coupling of CH_4_ to C_2_H_6_ and C_2_H_4_ [79].

**Figure 5:**
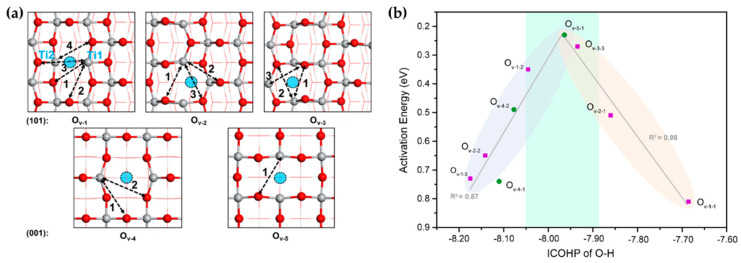
(**a**) Potential FLPs assembled by Ti and O on anatase TiO_2_(101) and TiO_2_(001) with O_v_s (denoted by blue circles). (**b**) Correlations between the integration of crystal orbital Hamilton population (ICOHP) of O–H with H_2_ activation energy, based on PBE-D3 via VASP. Adapted from reference [80] with permission.

We noticed a recent DFT study by Lin’s group showing that Ti and O sites on anatase TiO_2_(100) and TiO_2_(110) surfaces can form diverse FLPs with different distances between Ti and O (Figure 5a) in the presence of oxygen vacancies [80]. These Ti–O FLPs can heterolytically cleave H_2_ to form Ti–H and O–H species, which then react with acetylene to produce ethylene. Interestingly, the calculation results showed a volcano-shaped relationship between the H_2_ activation energy and the strength of the O–H bond (Figure 5b), and the barriers of the first and second steps of acetylene hydrogenation are positively correlated with the strength of Ti–H bonds and C_2_H_3_ adsorption energy, respectively. Importantly, this theoretical prediction of the hydrogenation reactivity of TiO_2_ was confirmed by an experiment [80], thereby providing useful insights for the future development of metal-oxide-based FLPs catalysts.

**Figure 6:**
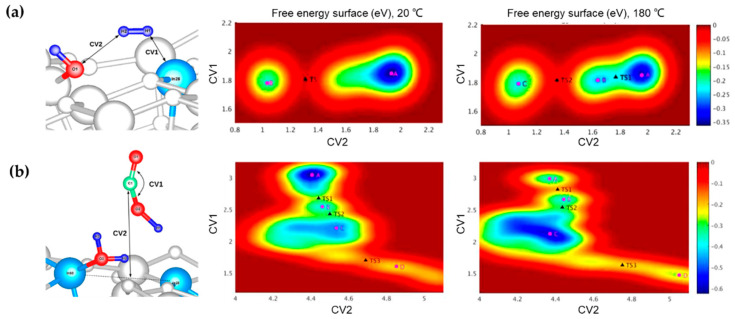
Collective variables (CVs) used in metaD AIMD simulations of the free energy surfaces of (**a**) H_2_ dissociation and (**b**) CO_2_ reduction catalyzed by In/O FLP at 20 and 180 °C. Color scheme: catalyst surface, white; LA (In), light blue; H, blue; O, red. Adapted from reference [81] with permission.

It is important to recognize that the FLP is a dynamic entity whose properties can be altered by external parameters such as temperature. Indeed, the impact of temperature on the surfaces of In/O FLPs with regard to catalyzing RWGS was investigated in the temperature range of 20 to 180 °C by using the metadynamics-biased ab initio molecular dynamics (metaD AIMD) [81]. The FLPs were found to be structurally altered at a high temperature (180 °C), as the distance between the LA and LB fluctuated by 0.04 Å. This led to a reduction of the barrier of the heterolytic H_2_ dissociation by 0.15 eV (based on Perdew−Burke−Ernzerhof (PBE) with Rappe−Rabe−Kaxiras−Joannopoulos (RRKJ), via Quantum Espresso (QE)) compared to that at 20 °C (Figure 6a). While the reduction of CO_2_, considered as the rate-limiting step, is not sensitive to temperature changes, the calculated energy barrier for the adsorption of CO_2_ at the FLPs is reduced by 0.19 eV (Figure 6b). The results highlight the important role of thermal fluctuation in the spatial separation of FLPs, which is closely related to the enhanced reactivity.

In another example, Fabris and workers computed the free energy surface for H_2_ activation on CeO_2_(111) using metadynamics [82]. Although no oxygen vacancy was included in the simulation, this study also demonstrated that the barrier (0.73 eV, based on PBE via QE combined PLUMED) is significantly reduced at the simulation temperature (350 K) relative to the 0 K result (0.99 eV), due apparently to the thermal fluctuation of the surface O species.

The temperature dependence of stable Ce–O FLPs on CeO_2_(110) was discussed by Huang et al., who reported that the formation of surface FLPs is dependent on the number of oxygen vacancies [79]. Although static DFT calculations indicated that the FLPs in the presence of fewer oxygen vacancies were unstable, AIMD results showed that the FLP sites can dynamically regenerate at high temperatures.

### 3.2. FLPs Based on Functionalized Surfaces

As discussed in the above section, heterogeneous FLPs on metal oxides are assembled from pre-existing Lewis acid and base sites. However, for most solid surfaces, the chemical environment is relatively simple and does not allow the natural formation of FLPs. Therefore, the introduction of foreign species as the LA or LB on the surface is an effective strategy for constructing surface FLPs. These studies not only provide us with alternative perspectives to understand the processes involved in heterogeneous catalysis, but also extend the application of heterogeneous FLP catalysis and design strategies for future FLP-based catalysts.

**Figure 7:**
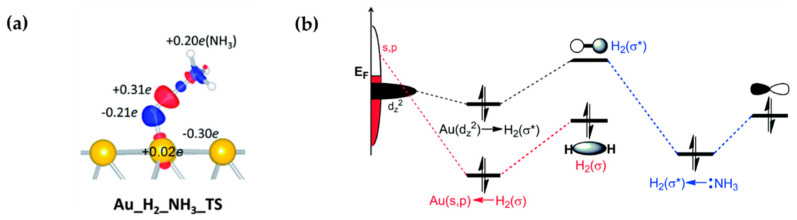
(**a**) Charge density difference map of the transition state of H_2_ activation catalyzed by the Au/NH_3_ FLP. (**b**) Schematic illustration of the favorable interactions among Au, H_2_, and NH_3_, symbol * indicated the antibonding orbital. Adapted from reference [50] with permission.

The simplest case would be pre-absorption of LAs and LBs on surfaces as the electron acceptor or donor, respectively. Lu et al. found that the pre-adsorption of imine or nitrile as LBs can enable efficient H_2_ activation on a modified gold surface, where the gold atom serves as the LA [50] (Figure 7a). The DFT calculation results suggested that the enhanced reactivity of the functionalized gold surface can be ascribed to the synergetic effects of the frustrated LA and LB sites, on which the partially filled s- and p-states of gold accept the electrons from the H_2_ σ orbital (Figure 7b). Similarly, Fiorio et al. reported that the pre-adsorption of nitrogen-containing Lewis bases on Au nanoparticles can also couple with surface Au atoms to generate heterogeneous FLPs [51,52]. Interestingly, they found that FLPs composed of Au LA sites and amine species with different basicity can be used to tune H_2_ heterolytic activation, which is consistent with the experimental observations. In addition, a reaction intermediate can also act as LB. For example, in a first-principles study, Jian et al. demonstrated that Ni_1_(OH)_2_/TiO_2_ possesses superior reactivity and selectivity for acetylene hydrogenation [83], in which the adsorbed C_2_H_3_ and C_2_H_5_ intermediates produced during the acetylene hydrogenation can serve as LBs to form FLPs with the surface Ni sites to accelerate the heterolytic H_2_ cleavage. The enhanced reactivity of metal surfaces by the introduction of foreign species to create new FLPs offers a useful strategy for the development of other metal-based hydrogenation catalysts.

Porous MOFs provide a flexible template and suitable nanospace to create FLPs. For example, Ye et al. reported that 1-(difluoroboranyl)-4-methyl-1H-pyrazole grafted in the MOF of UiO-66 exhibits the characteristics of an FLP formed by P (LB) and B (LA) atoms [84]. From first-principles calculations, the authors found that this FLP has the capacity to catalyze the heterolytic H_2_ dissociation to form P–H and B–H species, the key step in CO_2_ reduction with H_2_. The calculated minimal energy reaction pathway indicates that the CO_2_ is hydrogenated with the pre-adsorbed H species at FLP sites. The heterogeneous FLP (UiO-66-P-BF2) showed higher reactivity than the homogeneous FLPs catalyst (1-[bis(pentafluorophenyl)boryl]-3,5-aditert-butyl-1H-pyrazole).

**Figure 8:**
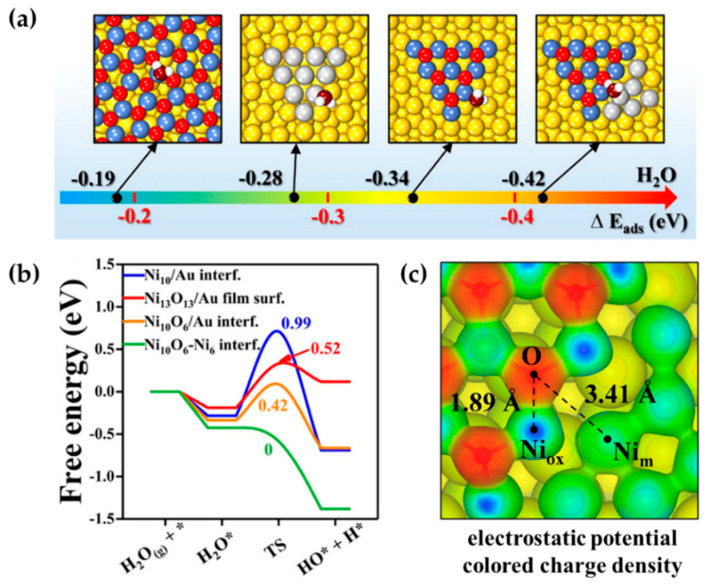
(**a**) Structures and adsorption of H_2_O on Ni_13_O_13_/Au(111), Ni_10_/Au(111), Ni_10_O_6_/Au(111), and Ni_10_O_6_-N_i6_/Au(111). Color code: Au, yellow; metallic Ni, grey; oxidized Ni, blue; oxygen in NiO_X_, red; oxygen in H_2_O, brown; hydrogen, white. (**b**) Gibbs free energy diagram of H_2_O dissociation on the four surfaces (* indicated the adsorption site), based on PBE with vdW-DF, via VASP. (**c**) Electrostatic potential colored electron density of Ni_1 0_O_6_-N_i6_/Au(111). Adapted from reference [85] with permission.

In addition, FLPs can also be created via engineering the interface between metal and metal oxide. For example, Zhao et al. reported that interfaces between metallic or oxidized nickel species and a Au surface (Figure 8a) are active for H_2_O dissociation [85]. Furthermore, their DFT results showed that the Ni_13_O_13_/Au(111), Ni_10_/Au(111), Ni_10_O_6_/Au(111), and Ni_10_O_6_-Ni_6_/Au(111) interfaces have different adsorption strengths for the adsorption of H_2_O (Figure 8a). Among them, the NiO_x_–Ni interface possessed the strongest adsorption of H_2_O, which is controlled by the Lewis acidity of the Ni site. In particular, H_2_O dissociation at the NiO_x_-Ni interface is a spontaneous process (Figure 8b) in which metallic Ni acts as an LA to accept the hydroxyl, while the O serves as the LB to capture a proton with the frustrated LA–LB distance of 3.41 Å (Figure 8c). This work points out that the heterogeneous FLPs can also be constructed at metal and metal oxide interfaces, extending the applications of FLP in heterogeneous catalysis.

Besides the surface FLPs mentioned above, FLPs based on nanostructured carbon materials have also been reported recently. Primo et al. demonstrated that graphene can hydrogenate the C_2_H_2_ to C_2_H_4_ with high conversion rates and selectivity in the absence of noble metals [86]. Interestingly, the addition of metal impurities (e.g., Mn and Pd) affects the hydrogenation performance little, and even sometimes decreases the reactivity. The comparison between the performance of graphene, graphene oxide, reduced graphene oxide, and N/P/S-contained graphene also suggested the reactivity originates from intrinsic sites of graphene. More interestingly, the reactivity of graphene was significantly and reversibly affected by the pre-adsorption of CO_2_ and NH_3_. Based on these observations, Primo et al. concluded that the hydrogenation reactivity can be ascribed to surface FLPs. However, the mechanism from the perspective of FLPs is still unexplained.

Of course, FLPs can also be created by substitutive doping, which changes the chemical structure of graphene. Sun et al. proposed an FLP catalyst for H_2_ activation by boron and nitrogen co-doping the bilayer graphene (BN-G) [87]. From DFT calculations, three BN-G configurations were obtained, including AA stacking (AA-01), AB stacking (AB-01), and AB stacking (AB-02). H_2_ activation in the interface between the two layers of these three structures was further investigated and compared with the process catalyzed by pristine bilayer graphene. The calculated activation energy of H_2_ dissociation on pristine bilayer graphene is 2.3 eV; it is 0.99, 1.05, and 1.36 eV on AA-01, AB-01, and AB-02, respectively (TPSS function with D2 via VASP). It was thus proposed that the FLP formed by B and N catalyzes the heterolytic H_2_ cleavage. Furthermore, Sun et al. found that dopants at the graphene edge possess higher reactivity towards H_2_ dissociation, and that H_2_ could spontaneously dissociate at the B site on the edge, revealing that the dopants at the edge could contribute more reactivity than the dopants in the basal plane. Very recently, Chen et al. argued that these B–N sites might not be responsible for these FLPs [56].

**Figure 9:**
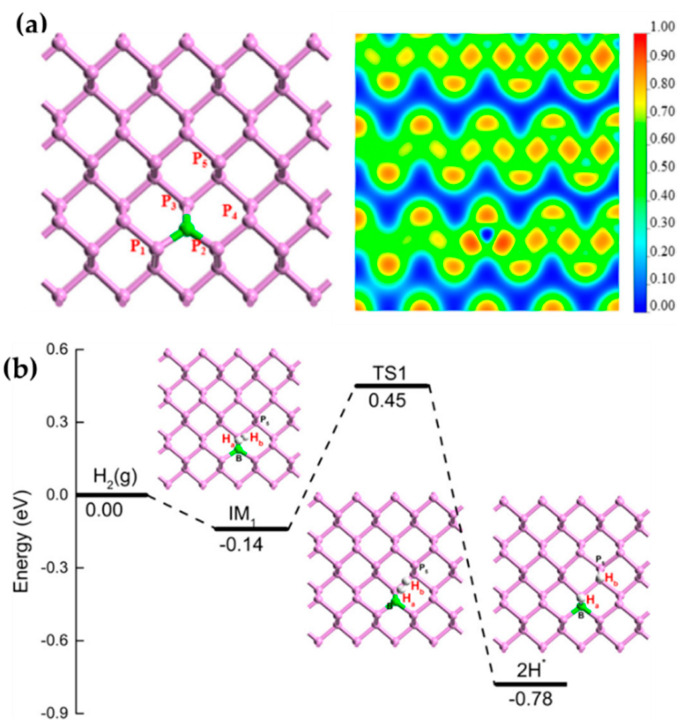
(**a**) Structure and electron localized function (ELF) diagram of B doped phosphorene. (**b**) Energy diagram of H_2_ activation catalyzed by B doped phosphorene, symbol * indicated adsorption site here. Adapted from reference [54] with permission.

Besides 2D carbon-based materials, other 2D structures have also been investigated for constructing FLP-based catalysts. Zhao et al. reported an FLP catalyst based on phosphorene via a doping strategy [54]. In particular, the B introduced to the phosphorene monolayer serves as the LA and P acts as the LB (Figure 9a, left). The charge transfer can be seen in the ELF results (Figure 9a, right): the electron density on the B atom is highly delocalized, indicating it is charge-deficient, whereas the electron density on the P atom is strongly localized, suggesting that it has lone pair electrons. H_2_ activation catalyzed by such an FLP would result in a much lower barrier of 0.59 eV (based on double numerical plus polarization basis set and PBE+D2, via DMol^3^) (Figure 9b) compared to 2.25 eV on pristine phosphorene, suggesting high activity of the newly formed FLPs for H_2_ activation. They also found that, using Me_2_C=O, HCN, and C_2_H_4_ as probe molecules, the H species produced over the FLPs can attack the unsaturated molecules to complete the hydrogenation catalysis.

Doping of 2D materials by metals can also lead to FLPs. Chen et al. reported transition metals and boron co-doped phosphorene with promising N_2_ reduction reaction (NRR) electrochemical performance [88]. Usually, it is well recognized that the electrocatalytic NRR performance can be improved by stabilizing the key N_2_H intermediates [89]. These authors demonstrated an alternative strategy based on the concept of FLPs. Accordingly, the B atom acts as the LA to provide an empty orbital, while the transition metal acts as the LB to donate electrons for enhancing N_2_H adsorption.

**Figure 10:**
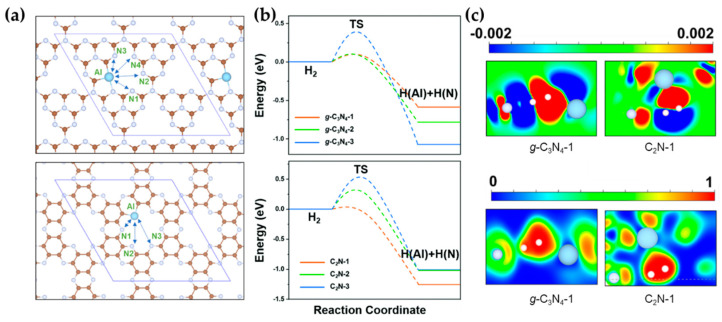
(**a**) Scheme of potential FLPs on Al-doped *g*-C_3_N_4_ (upper) and C_2_N (bottom). (**b**) Energy profile of H_2_ activation on Al/N FLPs. Based on PBE-D2 via VASP. (**c**) CDD (upper) and ELF (bottom) maps for TSs of H_2_ activation on *g*-C_3_N_4_-1 and C_2_N-1. Adapted from reference [55] with permission.

The carbon nitride group is another 2D material that is widely used in heterogeneous catalysis [90]. For example, Wan et al. reported Al/B doping C_2_N and *g*-C_3_N_4_ to construct FLPs from their DFT calculations [55]. These theoretical results showed that Al–N FLPs were more active than B–N FLPs for H_2_ activation. Introducing Al as the LA breaks the electron-rich environments at the pore edge and forms several Al–N combinations with varying electronic structures as FLPs (Figure 10a). These FLPs could efficiently activate H_2_ to form Al–H and N–H (Figure 10b), which can then hydrogenate C_2_H_2_ to C_2_H_4_. Similarly to transition states on defect CeO_2_(110) (Figure 4b), CDD and ELF maps in Figure 10c suggest the electron transfer between H_2_ with the Al/N FLP. Both cases utilize the intrinsic LB sites of carbon nitride, taking advantage of the introduced electron-deficient LA, to achieve the small molecule activation in the absence of noble metals.

## 4. Conclusions and Prospects

Significant efforts have recently been made to explore the applications of heterogeneous FLP catalysts for various reactions. Herein, we provided a comprehensive review of theoretical understanding of the mechanisms of FLP-facilitated heterogeneous catalysis. The insights provided by these theoretical results, coupled with experimental observations, offer a framework for in-depth understandings of heterogeneous FLP catalysis and design principles.

The key to heterogeneous FLP catalysis is the presence of spatially separated but adjacent surface active sites with electron-rich and deficient properties, respectively. The geometric separation of these LA and LB sites is important because it prevents direct bonding that would neutralize them, as in a CLP, while offering a suitable cavity for the cleavage of the targeted bond. Hence, the optimization of the separation between the FLP sites and the acidity/basicity is essential for the catalyzed activation of molecules, such as H_2_ and CH_4_. The adjacent LA and LB sites also polarize the molecule, resulting in a lowered dissociation barrier. In the H_2_ case, for example, its dissociation results in the formation of protonic and hydridic species, which can then be used to carry out subsequent reaction steps.

The heterogeneous FLP systems reported so far can be generally divided into two categories based on materials. One class is based on metal oxides, and the other relies on functionalizing surfaces. The former has abundant LA(M) and LB(O) sites of its own and is subjected to modifications by external means, such as substitutive doping. The doped metal can either serve as the LA or help to create an LA site by promoting oxygen vacancies. Alternatively, one can also control the LB site by hydroxylation. On the other hand, the latter type relies on the introduction of LA and LB species through functionalization. This can be readily achieved in porous materials such as MOFs, where the distance between the LA and LB sites can be readily controlled. The functionalization can also take place on metal surfaces, where the introduction of LB species transforms metal atoms as LA sites. Non-metal FLPs can be created analogously by doping 2D materials such as graphene. These strategies provide a range of tools for designing FLPs in heterogeneous systems.

The rapid development in the field serves as a manifestation of the powerfulness and universality of the FLP concept in heterogeneous catalysis. We expect more flexible ways to create FLPs in the future with traditional and novel LAs and LBs. Materials, such as metal-support interfaces and corrugated/reconstructed 2D materials, possessing regions with different electron gaining and losing abilities, might be leveraged to generate potential FLPs. Due to its outstanding performance in activating small molecules and the avoidance of noble metals, FLPs have prospects in wider applications in the future for heterogeneous catalysis. We expect robust future development in this field.

## Data Availability

Not applicable.
